# Zearalenone (ZEN) and Its Influence on Regulation of Gene Expression in Carp (*Cyprinus carpio* L.) Liver Tissue

**DOI:** 10.3390/toxins9090283

**Published:** 2017-09-15

**Authors:** Constanze Pietsch

**Affiliations:** Institute of Natural Resource Sciences (IUNR), Zurich University of Applied Sciences (ZHAW), Grüental, P.O. Box, CH-8820 Waedenswil, Switzerland; constanze.pietsch@zhaw.ch

**Keywords:** mycotoxin, immune system, gene expression, aquaculture

## Abstract

Zearalenone (ZEN) is a frequently-occurring mycotoxin in both animal and fish feeds. In order to characterize its effects on carp, three groups of fish were fed for 28 days with feeds contaminated with three different levels of ZEN (low: 332 µg kg^−1^, medium: 621 µg kg^−1^, and high: 797 µg kg^−1^ feed). The reversibility of the effects of ZEN was assessed by feeding all of the groups with uncontaminated feed for a further 14 days. Gene expression of immune genes in the liver tissue of the fish was analysed, revealing reduced expressions of immune, antioxidative, and estrogen-related genes after the fish had been exposed to ZEN. However, the expression of vacuole-type H^+^ ATPase increased substantially with ZEN exposure, thus supporting the previously-reported sensitivity of lysosomal functions to ZEN. Feeding the fish with a ZEN-free diet for a further two weeks changed the effects of ZEN on the expression of some genes, including the expressions of the cytokines IL-1β, IL-8, IL-10, and arginase 2, which were not influenced after four weeks of treatment, but showed lower values after the recovery phase in fish previously treated with ZEN compared with the control group. In summary, this study confirmed the broad effects of ZEN on different essential functions in carp and suggests that the current maximum allowable levels in compound feed are too high to prevent damage to fish.

## 1. Introduction

The mycotoxin zearalenone (ZEN)frequently occurs in feed stuffs and ingredients [1,2,3]. In addition to hepatotoxic, genotoxic, haematological, and reproduction effects [4,5,6,7,8] of ZEN on fish, concern relating to the effects of this fungal metabolite on the immune system is also increasing.

Many mycotoxins are assumed to be immunotoxic in higher vertebrates [9]. For example, dietary exposure to ZEN has been shown to impair immune functions in domestic and laboratory animals [10,11,12,13,14]. This has included impairment of both innate and acquired immune responses [15,16]. However, the effects of ZEN on the immune parameters of fish have rarely been investigated. Recent publications have reported changes in white blood cell populations [7] and immunomodulation in carp treated with ZEN for four weeks [8], but the mechanism of action of ZEN remains unknown. The present study intends to expand knowledge on the effects of this mycotoxin on the immune system of fish. The initiation of inflammatory responses is characterized by pro-inflammatory cytokines such as IL-1β, TNFα, and IFNγ. In addition, IL-8 is an inducible pro-inflammatory cytokine that leads to migration of immune cells to the site of inflammation [17,18]. Pro-inflammatory cytokines often lead to the production of high quantities of antimicrobial nitric oxide (NO) by activation of inducible NO synthases (iNOS) in fish phagocytes, which is typical for early pro-inflammatory reactions [19,20]. Tight regulation of inflammatory processes also includes regulatory mechanisms such as the synthesis of arginase by the leukocytes [21]. Arginases reduce the availability of arginine for NO production [22] and are involved in tissue repair [23,24]. Two isoforms of arginase that show tissue distribution and expression levels [25,26] are present in fish. Regulation of inflammatory responses in fish is also mediated by cytokines, such as TGFβ and IL-10 [27,28].

The investigation of pro-inflammatory or anti-inflammatory immune reactions is crucial since their interactions determine the outcome of immune responses. Contaminants in fish feed may have immune-modulatory consequences in fish, which may result in increased susceptibility to infectious agents in aquaculture settings. Hence, it may also be assumed that the presence of ZEN in feed, which is an immune-modulating agent in fish [8], is detrimental to fish health and immunity.

ZEN is known as a potent mycoestrogen that exhibits similar in vitro affinity for both forms of mammalian estrogen receptors, ERα and ERβ [29]. It is assumed that ZEN has an estrogenic potency in fish of approximately 50% compared with that of natural 17β-estradiol [30]. However, ZEN has exhibited toxic effects in fish cell lines with non-detectable nuclear ER expression [6], which casts doubt on the assumption that ZEN effects in cells are generally mediated by this pathway.

To further understand the cellular mechanisms that are involved in ZEN toxicity the mRNA expression of anti-oxidative and lysosomal enzymes and estrogen-regulated genes were also investigated. There were two reasons for this. Firstly, lysosomal activity has been reported to be sensitive to ZEN toxicity in permanent fish cell lines [6]. Secondly, ZEN, a potent estrogenic compound [31], has not been shown to induce a vitellogenin response at the protein level in juvenile carp treated with ZEN-contaminated diets [7]. To improve our mechanistic understanding of ZEN actions, this lack of effect made it necessary to evaluate whether changes of gene expression levels are present despite the absence of effects at the protein level. For comprehensive investigations of estrogenic effects in carp, not only vitellogenin expression was evaluated, but also the mRNA expression of estrogen receptors and transferrin. The latter is known to be regulated by estrogens and is also connected to disease resistance in carp [32,33].

To expand our knowledge of the systemic actions of ZEN in fish, the present study examined the effects of ZEN on the mRNA expression of selected genes in carp (*Cyprinus carpio*), which is an important fish species for aquaculture [34].

## 2. Results

### 2.1. Effects on mRNA Expression of Immune Genes

**Figure 1:**
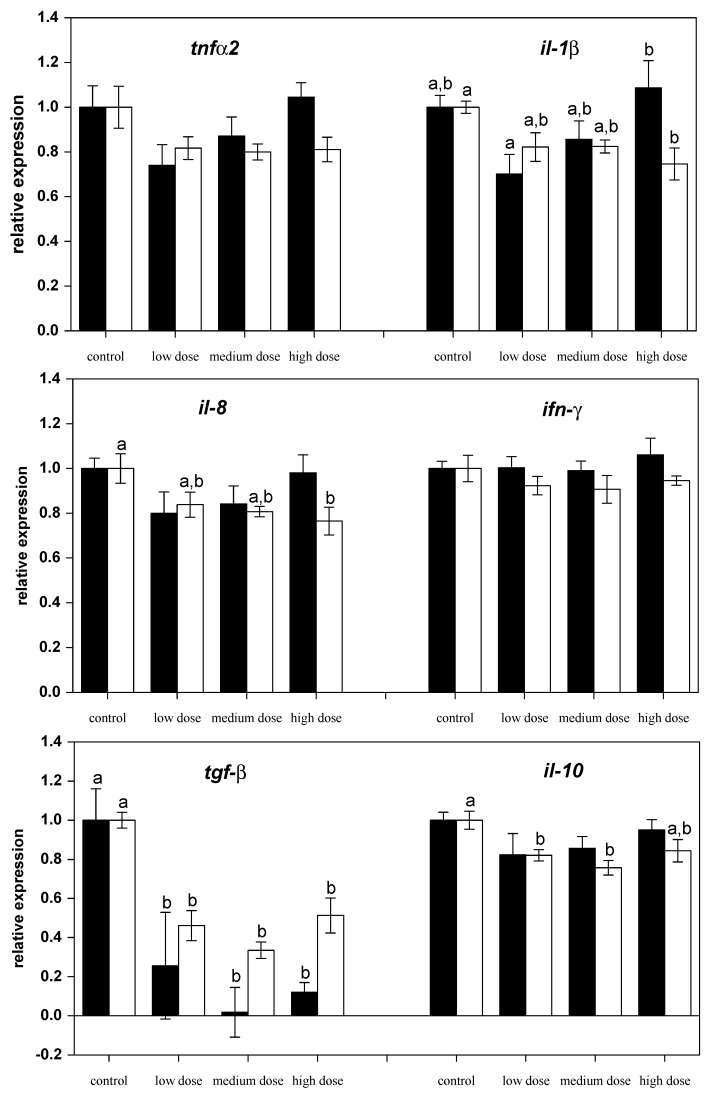
Relative mRNA expressions of pro-inflammatory and anti-inflammatory cytokines in liver of fish treated for four weeks with three different concentrations of ZEN (low: 332 µg kg^−1^, medium: 621 µg kg^−1^, and high: 797 µg kg^−1^ feed) compared to untreated control fish (black bars), including an additional two weeks of recovery (white bars). All samples were analyzed in duplicate per fish. The mean ± SEM of six fish per sampling and treatment group is shown; means with the same letter (a and/or b) are not significantly different from the control fish (significance tested with Mann-Whitney U-tests, *p* = 0.05).

**Figure 2:**
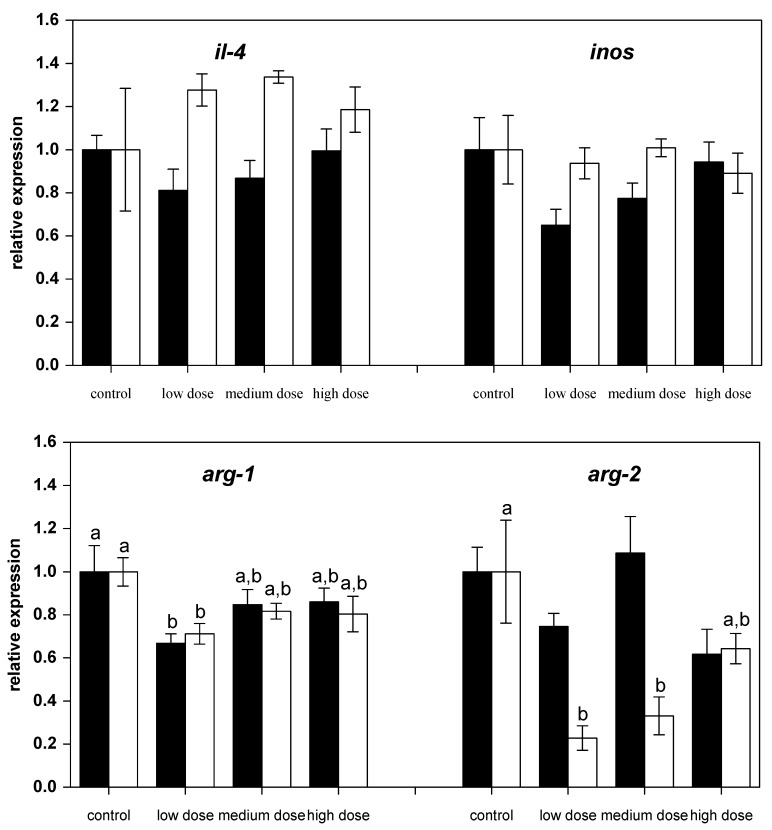
Relative mRNA expressions of pro-inflammatory and anti-inflammatory immune genes in liver of fish treated for four weeks with three different concentrations of ZEN (low: 332 µg kg^−1^, medium: 621 µg kg^−1^, and high: 797 µg kg^−1^ feed) compared to untreated control fish (black bars), including an additional two weeks of recovery (white bars). All samples were analyzed in duplicate per fish. The mean ± SEM of six fish per sampling and treatment group is shown; means with the same letter (a and/or b) are not significantly different from the control fish (significance tested with Mann-Whitney U-tests, *p* = 0.05).

The investigation of mRNA transcript levels in liver showed differential regulation of genes. The relative mRNA levels of pro-inflammatory (*il-1β*, *ifnγ1*, *tnfα-2*, *il-8*, *inos*) and anti-inflammatory (*il-10*, *il-4*, *arg-1* and *arg-2*) immune-related genes were assessed in liver from control and ZEN-fed carp after 28 days of experimental feeding (Figure 1 and Figure 2). Exposure of fish to different ZEN doses in their feed did not result in significant changes to the gene expression of *tnfα-2* and *il-1β* compared to the control fish (Figure 1).

However, after two weeks of recovery, mRNA expression of *il-1β* and *il-8* in fish treated with the high dose of ZEN was lower than for the control fish (Figure 1). The mRNA levels of the anti-inflammatory gene *tgf-β* were low in all ZEN-treated fish and remained low even after the recovery phase. The mRNA expression of *il-10* in fish recovering from being treated with the low and medium ZEN doses, but not in the animals exposed to the high dose of ZEN, was also lower than in control animals. However, the expression of *ifn-y* (Figure 1) and *il-4* (Figure 2) was not influenced by ZEN feeding. The expression of the pro-inflammatory enzyme *inos* was not influenced by the ZEN treatment, but the expression of the anti-inflammatory enzyme *arg-1* was reduced in fish treated with the low dose ZEN even after the recovery phase. In contrast, mRNA levels of *arg-2* were found to be reduced only after the recovery phase in fish that had previously been treated with the low and medium ZEN doses (Figure 2).

### 2.2. Effects on mRNA Expression of Estrogen-Sensitive Genes

**Figure 3:**
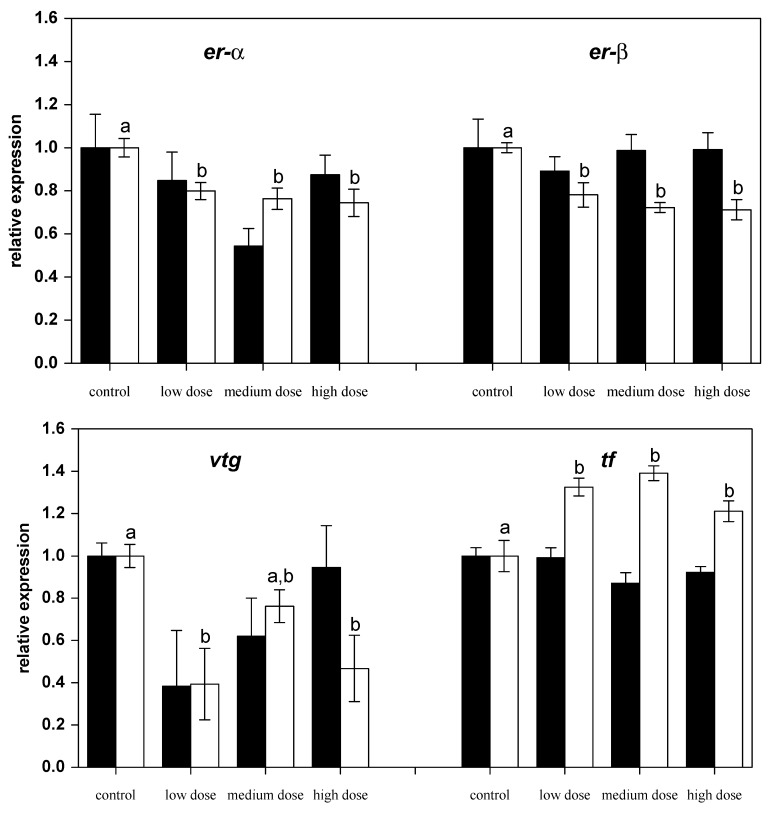
Relative mRNA expressions of estrogen-related genes in liver of fish treated for four weeks with three different concentrations of ZEN (low: 332 µg kg^−1^, medium: 621 µg kg^−1^, and high: 797 µg kg^−1^ feed) compared to untreated control fish (black bars), including an additional two weeks of recovery (white bars). All samples were analyzed in duplicate per fish. The mean ± SEM of six fish per sampling and treatment group is shown; means with the same letter (a and/or b) are not significantly different from the control fish (significance tested with Mann-Whitney U-tests, *p* = 0.05).

After 28 days of exposure to ZEN, the mRNA expression of the nuclear estrogen receptors *er-α* and *er-β2* had not changed compared to the control fish, but after the recovery phase expressions of both genes were found to be lower in the fish previously treated with ZEN (Figure 3). Similarly, the mRNA levels of estrogen-regulated gene for the egg yolk precursor vitellogenin (*vtg*) in fish pre-treated with the low and the high ZEN doses were noted to be lower after the recovery phase. The mRNA levels of the iron-binding protein transferrin (*tf*) were found to be higher in all of the ZEN-treated fish after the recovery phase.

### 2.3. Effects on mRNA Expression of Anti-Oxidative Genes

**Figure 4:**
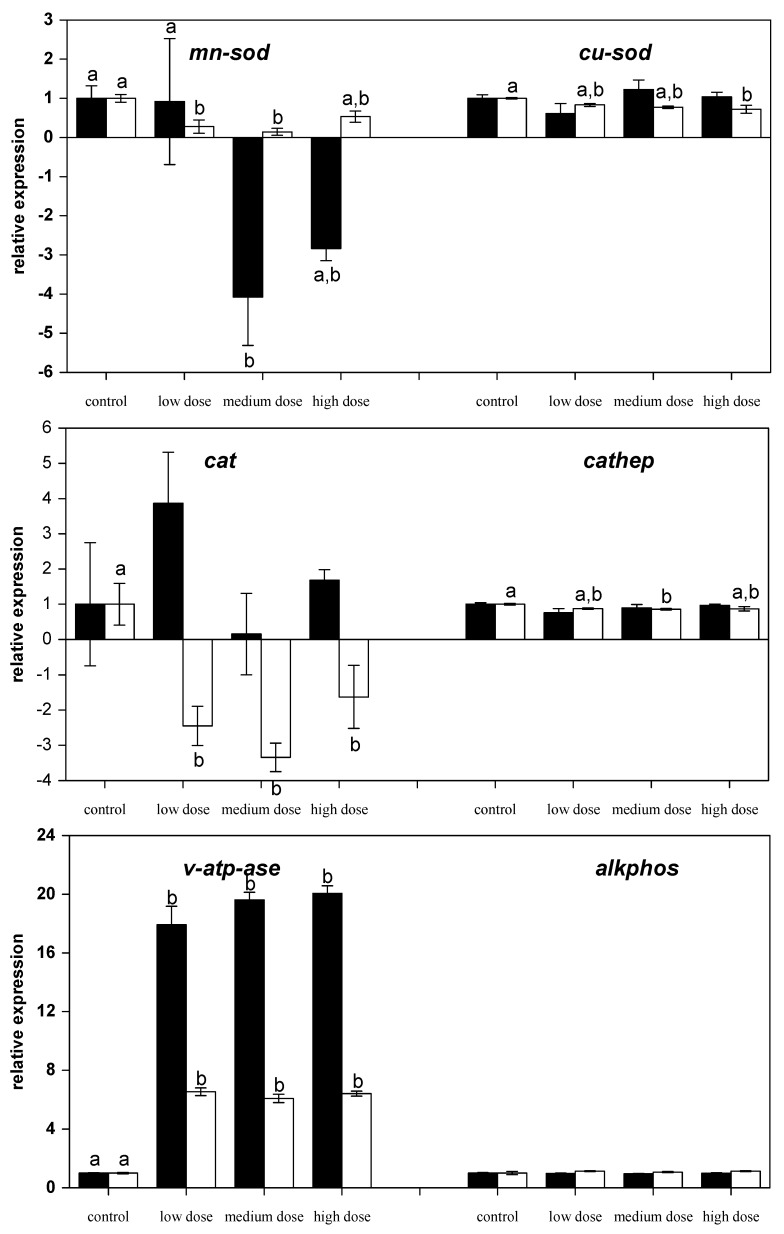
Relative mRNA expressions of anti-oxidative and lysosome-related in liver of fish treated for four weeks with three different concentrations of ZEN (low: 332 µg kg^−1^, medium: 621 µg kg^−1^, and high: 797 µg kg^−1^ feed) compared to untreated control fish (black bars), including an additional two weeks of recovery (white bars). All samples were analyzed in duplicate per fish. The mean ± SEM of six fish per sampling and treatment group is shown; means with the same letter (a and/or b) are not significantly different from the control fish (significance tested with Mann-Whitney U-tests, *p* = 0.05).

In contrast, the mRNA levels of the anti-oxidative and manganese-dependent enzyme superoxide dismutase (*mn-sod*) were reduced by medium-dose ZEN feeding for four weeks and also reduced in the fish previously fed the low and medium ZEN feed after the recovery phase (Figure 4). In contrast, mRNA levels of the copper-dependent isoform of the superoxide dismutase (*cu-sod*) were only found to be reduced after the recovery phase in fish that were previously treated with the high-dose ZEN diet. The mRNA expression of anti-oxidative enzyme catalase (*cat*) was also reduced in ZEN-fed fish after the recovery phase.

### 2.4. Effects on mRNA Expression of Lysosomal Genes

The lysosomal acidic endoproteinase cathepsin D (*cathep*) mRNA expression was found to be lower in fish fed the medium-dose after the recovery phase of an additional two weeks (Figure 4). The mRNA expression of the v-type H^+^ ATPase (*v-atp-ase*) was ~17- to 20-fold higher in the liver of all the ZEN-fed carp compared to the control-fed carp. Even after a recovery phase of two weeks the mRNA expression of this gene was still six-fold higher in the ZEN-fed fish than the expression in the control fish. In contrast, the hydrolytic enzyme alkaline phosphatase (alkphos) was not influenced by ZEN feeding.

## 3. Discussion

### 3.1. Immunotoxicity of ZEN and Anti-Oxidative Responses

Mycotoxins, such as ZEN, are assumed to primarily exhibit their cytotoxic potential on liver tissue of mammals [35,36], but our understanding of its effects and mode of actions is insufficient in fish. Immunotoxicity of ZEN has previously been investigated in carp, but the reason for the immunomodulative effects of this mycotoxin remained unknown [8]. The present study confirms the immunomodulative potential of ZEN in carp liver tissue and reveals mostly immunosupressive effects. Relevant studies on the effects of ZEN on inflammatory cytokines have revealed controversial results, showing that ZEN and its derivatives either induce [37,38] or suppress [10,11,15,39,40] the expression of pro-inflammatory cytokines. There are several reasons for the, to date, inconclusive assumptions about the reasons for the immunotoxic potential of ZEN and the involvement of oxidative stress in the toxicity of ZEN in fish and other vertebrates.

Firstly, the effects of ZEN on fish and fish cells involve oxidative stress similar to higher vertebrates [6,7]. Surprisingly, if the mRNA expression of anti-oxidative enzymes in liver tissue was changed, then it was found to be lower than the levels of the control fish and there was no increase in order to compensate for potentially increased cellular oxidative stress. Activity of superoxide dismutases (SOD) in fish livers is known to increase after short-term exposure to toxic substances but prolonged exposure indicated that SOD activity gradually decreases [41]. The lack of response in terms of mRNA expression of Cu/Zn-SOD compared with the expression of Mn-SOD in the present study is in accordance with several mammalian studies that exposed animals to oxidants [42,43,44]. In addition, SOD expression has been shown to be temperature-sensitive in different fish species [45,46], and it is assumed that changes of SOD activity in fish may also reveal general stress responses. It has also been previously reported that exposure of permanent fish cell lines to ZEN leads to differences in the production of reactive oxygen species [6]. Feeding carp with low ZEN concentrations also led to increased respiratory burst in carp leukocytes, whereas high ZEN levels had an immunosuppressive effect [8]. Similarly, different effects were observed in the present study using three ZEN concentrations in the feed on immune gene expression in liver (Figure 1). Thus, differential, dose-dependent effects of ZEN were confirmed. A contribution of ZEN accumulation in fish to this dose-dependent effects can be mostly excluded due to the fact that the concentrations of ZEN and its metabolite α-zearalenol (α-ZEL) were found to be low in fillet of the experimental fish [7]. This fact is also supported by the rapid metabolization of ZEN by fish cells in vitro [6].

Secondly, it is clear that species-specific responses to ZEN exist, which make drawing of general conclusions about the immunotoxicity of ZEN more complicated. Examples may be the exposure of chicken splenic lymphocytes to ZEN, which led to reduced *ifn-γ* expression [13], or swine polymorphonuclear leukocytes, which showed reduced tnf-α expression due to ZEN treatment [11]. These cytokines did not change in the carp as a result of the ZEN treatment and the reasons for these differences remain obscure.

Thirdly, ZEN treatment caused an increase in immune responses in blood immune cells, whereas liver cytokine expression seemed to be suppressed [47]. This organ-dependent response to ZEN might also explain the difference in cellular responses of carp immune organs to ZEN [8] compared with the responses that were observed in liver tissue in this study.

Some cell-types show mixed responses to ZEN, including concurrent pro-inflammatory and anti-inflammatory responses and, thus, may not be explained by the above-mentioned influencing factors on ZEN toxicity. For example, ZEN exposure caused the gut-associated immune systems of swine to exhibit different effects in terms of pro-inflammatory and anti-inflammatory cytokines [48]. Similarly, high ZEN doses reduced mRNA expression of pro-inflammatory genes (*il-1β*, *il-8*) and some anti-inflammatory cytokines (*tgf-β* and *il-10*) after the recovery phase in the present study. However, there was no reduction observed in *il-4* levels. Similar results were reported in studies investigating spleenic and hepatic responses to ZEN in swine [38,49]. In the present study, the mRNA expression of effector enzymes showed that iNOS was not influenced, but the expression of arginases was reduced by ZEN. This indicates a reduction of pro-inflammatory and antiinflammatory responses in carp. Thus, it can be assumed that ZEN impairs the inflammatory response and might affect the capacity of fish to eradicate potential infectious stimuli and to initiate appropriate healing processes. This can be especially problematic under husbandry conditions for fish in aquaculture.

### 3.2. Estrogenic Effects of ZEN

Livers in fish are important target organs for natural estradiol, and the synthesis of the yolk protein vitellogenin by hepatocytes cells is under the strict control of 17β-estradiol. However, the expression of ERα and ERβ2, which is usually high in carp livers [50], was found to be reduced after exposing the fish to ZEN. It is known that ZEN competes with natural estrogen for the ER binding sites in the uterus of rats [51]. However, and in contrast to higher vertebrates, detailed data on the effects of phyto- and mycoestrogens on the expression of the various ER subtypes in fish is lacking and knowledge of endogenous estrogens is very limited. From zebrafish, it is known that both receptors are differently regulated by 17β-estradiol, whereby ERα expression is strongly induced, but ERβ2 expression remains unchanged in the liver [52]. The sensitivity of ER subtypes to natural estrogen has been described as comparable in carp [50]. For certain ER subtypes, the mixed agonist-antagonist action of ZEN has been described [29] which might explain the unexpected effect of ZEN on ER expression. Rapid metabolization of ZEN in carp might also have contributed to the absent estrogenic responses, since it has been reported that metabolites of ZEN are only partial agonists of ER in zebrafish [53].

The production of vitellogenin which is essential for embryo development is tightly coupled to substantial estradiol-dependent upregulation of ER expression. In parallel to the decreased expression of ERs, vitellogenin expression was reduced in fish recovering from feeding on the low and the high ZEN diet and, thus, corresponds to the previously reported absent increase of the vitellogenin protein in serum after ZEN treatment in carp [7].

Only the transferrin subtypes D and G were investigated in the present study using the primers presented in Table 1 since they are the variants expressed in the carp that were used (transferrin typing is provided as supplementary data). Transferrin expression was found to increase in all fish recovering from ZEN exposure, but not directly after four weeks of exposure to ZEN.

### 3.3. Effects of ZEN on Expression of Lysosomal Genes

The effects on mRNA expression levels of lysosomal enzymes yielded insights into the cellular effects of ZEN in carp. Additionally, the fact that ZEN acts on endoplasmic reticulum function [55], which may at least in part explain its cytotoxic potential, lysosomes are closely related to the endoplasmic reticulum since both belong to the endomembrane system that is connected by vesicular transport. The pronounced effect of ZEN on the v-type H^+^ ATPase has not been previously described. In cells, the hydrolysis of ATP by ATPases is used to energize ion-transport processes across cell membranes. Hence, v-type H^+^ ATPases participate in the acidification of endosomal compartments of cells and transport processes across cell membranes and entire epithelia [56]. The lysosomal effects of ZEN on permanent fish cells have been previously described [6]. These effects might be due to a direct influence on v-type H^+^ ATPases which probably increases their expression to compensate for impairment at the protein level or due to indirect effects caused by cellular disturbance of the acid-base balance. Inhibition of v-type H^+^ ATPase function has to date been reported to be inhibited by dicyclohexylcarbodiimide, NO_3_^−^, *N*-ethylmaleimide, 7-chloro-4-nitrobenz-2-oxa-1,3-diazole, certain antibiotics, and the insecticidal mycotoxins destruxin and efrapeptin [57,58,59]. Although, the exact molecular mechanisms remain unknown, it is important to note that ZEN interferes with intracellular ion transport in cellular compartments.

In higher vertebrates, it was assumed for a long time that cellular distribution of cathepsin D is restricted to the acidic milieu of lysosomes where these enzymes participate in the non-specific protein degradation and play an important role in the regulation of apoptotic events [60]. Its function in activation and degradation of chemokines and growth factors, in apoptosis and in tumor progression [60] may play an important role in toxicity of xenobiotics, but its importance in fish cells has not yet been established. ZEN has previously been shown to have genotoxic effects in fish cells [6,7] and is a carcinogenic substance in mammals [61]. It may be assumed that reduced *cathep* activity is advantageous for cells treated with ZEN since this can be involved in reduction of cancer growth [60]. Thus, the changes of *cathep* expression in fish exposed to the medium dose ZEN may indicate an involvement of this enzyme in the toxicity of ZEN, but its full meaning has yet to be understood.

Alkaline phosphatase is a membrane-associated enzyme that is known to play a role in nutrient uptake and wound healing in fish [62,63]. Induction of alkaline phosphatase activity has also been reported in fish livers after exposure to toxic cyanobacteria [64], however, exposure of carp to ZEN did not influence the expression of this enzyme in liver tissue.

Finally, the exact cellular mechanism(s) of action of ZEN on cellular functions in different organs still remain to be investigated and further research is needed to provide an organ-wide description of possible effects of this mycotoxin on gene expression patterns in fish.

## 4. Conclusions

The low ZEN dose in the present study is comparable to ZEN values that can be found in commercially-available fish feeds [2]. ZEN feeding clearly affected the expression of immune genes, of antioxidative and lysosomal genes, and also influenced genes that are known to be estrogen-regulated at mycotoxin concentrations in the experimental diets that remained far below the maximum allowable level currently recommended by the European Commission [65]. This demonstrates that fish are at least as sensitive to ZEN as higher vertebrates [12,13,14,15,66,67]. Therefore, maximum allowable ZEN levels should be reconsidered for fish in aquaculture.

## 5. Materials and Methods

### 5.1. Chemicals

All chemicals were obtained from Sigma-Aldrich (Buchs, Switzerland) unless indicated otherwise.

### 5.2. Preparation of Feeds

The experimental diets were prepared without cereal ingredients to avoid exposure to more than one mycotoxin. Therefore, fishmeal, blood meal, casein, dextrose, potato starch, fish oil, vitamins and minerals were used to obtain four separate feeds that showed no nutritional differences [7]. Pelletization was repeated three times for each feed to guarantee for homogenous dispersal of all ingredients. The final diets were isonitrogenous (mean crude protein of 46.7%) and isocaloric (mean energy content of 22.6 MJ kg^−1^ dry matter). Confirmation of experimental ZEN enrichment in three of the feeds (low dose: 332 µg kg^−1^, medium dose: 621 µg kg^−1^ and high dose: 797 µg kg^−1^ final feed) was achieved by HPLC with fluorescence detection as described previously [7]. The control diet was not enriched with ZEN and, consequently, no ZEN was detected [7].

### 5.3. Exposure of Fish

The Aischgründer strain of mirror carp (Bavaria, Germany) were 12–16 cm in length at the start of the feeding trial. A regular light regime and a constant temperature of 24.9 ± 0.4 °C (mean ± SD) were maintained during the trial [7]. Fish were reared in 54 L tanks, each containing six fish in a flow-through system. Three groups of fish were fed the experimental ZEN-contaminated diets, while the control group received the uncontaminated feed at a feeding intensity of 3% of body weight per day. After four weeks of feeding, half of the fish were sampled (six fish per treatment group), and two additional tanks per treatment were fed the ZEN-free diet for two more weeks before the final sampling of six fish per treatment group. This was necessary to investigate possible recovery from ZEN feeding. After the indicated time of exposure, the fish were killed by a blow to the head, and the liver was surgically removed. The entire experimental procedures have been approved under permission number 2410 by the according Cantonal veterinarian authorities of Basel-Stadt (Switzerland).

### 5.4. Gene Expression Analyses

For gene expression analyses, liver tissue was immediately placed in RNAlater^®^ (Fisher Scientific, Reinach, Switzerland) for 24 h at 4 °C followed by storage at −20 °C until use. Total RNA was isolated from the samples using an RNeasy^®^ Plus Mini Kit (Qiagen AG, Hombrechtikon, Switzerland). Subsequently, two micrograms of RNA were reverse transcribed into cDNA using a High-Capacity cDNA Reverse Transcription Kit (Applied Biosystems, distributed by Life Technologies Europe B.V., Zug, Switzerland) according to the manufacturer’s instructions.

Thereafter, two micrograms of the obtained cDNA were diluted 1:20 (*v:v*) in nuclease-free water (Ambion^®^, distributed by Life Technologies Europe B.V., Zug, Switzerland) and used for real-time PCR using the Thermo Scientific ABsolute qPCR SYBR^®^ Green ROX Mix (Fisher Scientific, Reinach, Switzerland). Primers for carp interferon-gamma 1 (*ifnγ1*), tumor necrosis factor alpha-2 (*tnfα-2*), interleukin-1 beta (*il-1β*), interleukin-4 (*il-4*), interleukin-8 (*il-8*), interleukin-10 (*il-10*), inducible nitric oxide synthase (*inos*), arginase 1 (*arg-1*), arginase 2 (*arg-2*); transforming growth factor beta (*tgf-β*), estrogen receptors alpha and beta 2 (*er-α and er-β2*), vitellogenin (*vtg*), transferrin (*tf*) covering type D and type G transferrin (since these were the transferrin types present in the experimental fish; see Figure S1); catalase (*cat*), manganese-dependent superoxide dismutase (*mn-sod*), AJ_492825.1; copper/zinc-dependent superoxide dismutase (*cu-sod*), cathepsin D (*cathep*), v-type H^+^ ATPase (*v-atp-ase*), alkaline phosphatase *(alkphos*), and beta 2 microglobulin (*b2m*) as a reference gene were obtained from Microsynth AG (Balgach, Switzerland). The respective primer sequences can be found in Table 1. The expression of *b2m* was not different between the treatment groups. All gene products were cloned, sequenced, and primer conditions have been validated for real-time PCR usage. A Roche LightCycler^®^ LC480 real-time PCR machine (Roche Diagnostics AG, Rotkreuz, Switzerland) was used for gene expression analyses. The PCR cycling conditions included the following steps: initial denaturation at 95 °C for 15 min, followed by 40 cycles at 95 °C for 15 s, 60 °C for 30 s, and 72 °C for 30 s. A melt curve step (95 °C for 5 s, 60 °C for 1 min, and 5 acquisitions per °C until 97 °C was reached using a ramp rate of 0.06 °C per s) was added at the end of all runs to ensure that the individual PCR reactions yielded a single melting peak. All samples were run in duplicate. The results are presented as the mean ± SEM of six fish per experimental group for each sampling date.

### 5.5. Statistics

The effects of ZEN treatment were determined by comparison of the obtained values of the treatment groups to the respective controls at each sampling date using non-parametrical Kruskal-Wallis tests followed by Mann-Whitney U-tests (SPSS 9.0 for Windows; SPSS Inc, Chicago, IL, USA, 1999). Differences between treatment groups were considered statistically significant when *p* < 0.05.

## Figures and Tables

**Table 1:** Primers used in this study.

Primers	Sense Sequence (5′-3′)	Anti-Sense Sequence (5′-3)
*ifnγ1*	TGAGCTTAAAGAATGTGTGGCCCAA	ACTCCATATGTGACGGCTTTTGGT
*tnfα-2*	AGGACCAAGACGTCGTGCAT	GGCTTGTAGCTGCCGTAGGA
*il-1β* *il-4* *il-8*	ACCTTGCAGTGCACCATTTGTGA TTTCTGGGCTGTCTGGTGCCAA TTATTTTGCTTTCAGGAATGAGTCTTAG	TGTTGCTCTGCACCATAGTCTT TTTCTTGTCAGTACGGAAATGCTCA CACACTCTCTATGTGTTTTCCAATGC
*il-10*	GAACGAGATCCTGCGCTTTT	TTGAGTGCAAGTGGTCCTTCTG
*tgf-β* *inos*	AATCCTTTACTACATCGGAAAAACGCC AACAGGTCTGAAAGGGAATCC	TGTTGGACAGTTGTTCGATTTTGGGC CATTATCTCTCATGTCCAGAGTCTCTTCT
*arg-1*	TGAGGAGCTTCAGCGGATTAC	CCTATTATTCCCACGCAGTGATG
*arg-2*	GGAGACCTGGCCTTCAAGCATCT	CTGATTGGCACGTCCAACT
*er-α* *er-β2* *vtg* *tf* *cat* *mn-sod* *cu-sod* *cathep* *v-atp-ase* *alkphos* *b2m*	GGAGAATGTGTCGAGGGGATGGCT TACAGTCCCGCTCTGCTGGGTTAC TTCTGTTGGCACACCAGTC AGCAAGTGCAAAGCYTCTTCTGGA AGGAAACAACACCCCCATCT TCAAGCGTGACTTTGGCTCA AGCTGGTGAAAATGGGGTTGC TCCATGTCTTTGCACTGCTG GTGCAGAAATCAAAAGATGTGATGGAC TTAAATGGGCCAAAGATGCAGGC TTCAGGTGTACAGCCATTTTCCCG	TCCAGCATGCACTGCACCATGAAG GTGGCGTAAGTTCTGCCCAACTCC AGCCAGGAACTTCTCCTTGATG TGAAAGCCCCATCATAGCCA AAACAGAAGCGCATCCCTGAT TGACATCTTCTCCTTCATCTTCTG CAGCATTTTGTCCACAATGTCAA TCATTTCTTCTCCATGTGCCTCT ATGGCGAAGTTGTCTGCACTGT TGTTGTTGTGACGATTCCCACTGA TGTTCTCTTTTCCGTACTCTCCG

NCBI accession numbers of the known sequences of carp (or closely-related fish species as indicated) that have been used for designing the primers using the Primer 3 software [54]: *ifnγ*: consensus sequence based on AB_376666.1, AB_376667.1, and GQ_168344.1; *tnfα-2*: based on the known sequence of *Danio rerio* (NM_212859.2); *il-1β*: AB_010701.1; *il-4*: AB_697619.1; *il-8*: EU_011243.1; *il-10*: AB_110780.1, JX_524551.1, and JX_524550.1; *tgf-β*: AF_136947.1; *inos*: AJ_242906.1; *arg-1* and *arg-2*: as published elsewhere [25]; *er-α*: AB_334722.1; *er-β2*: AB_334724.1; *vtg*: consensus sequence derived from AB_331884.1, AF_414432.1, AB_089796.1, and AB_106873.1; *tf*: consensus sequence derived from type D and type G transferrin (EU_715323.1 and EU_715325.1); *cat*: GQ_376154.1; *mn-sod*: AJ_492825.1; *cu-sod:* consensus of the know sequences of *Hypophthalmichthys nobilis* (HM_469964.1, HM_469965.1, and HQ_008861.1); *cathep*: based on the sequence on cathepsin D from *Danio rerio* (BC_154315.1); *v-atp-ase*: JX_570880.1; *alkphos*: JF_411614.1; *b2m*: AM_690441.1.

## Supplementary Materials

The following are available online at www.mdpi.com/2072-6651/9/9/283/s1, Figure S1: Gelelectrophoretic separation of serum samples from carp belonging to the present study.
