# Supplementary material for: Zearalenone (ZEN) and Its Influence on Regulation of Gene Expression in Carp (Cyprinus carpio L.) Liver Tissue
Source: Toxins (Basel). 2017 Sep 15;9(9):283. doi: 10.3390/toxins9090283 (PMC5618216; doi:10.3390/toxins9090283)
Supplement: Supplementary file 1 [file toxins-09-00283-s001.pdf]

Supplementary Materials: Zearalenone (ZEN) and Its Influence on Regulation of Gene Expression in Carp (*Cyprinus carpio* L.) Liver Tissue

Constanze Pietsch

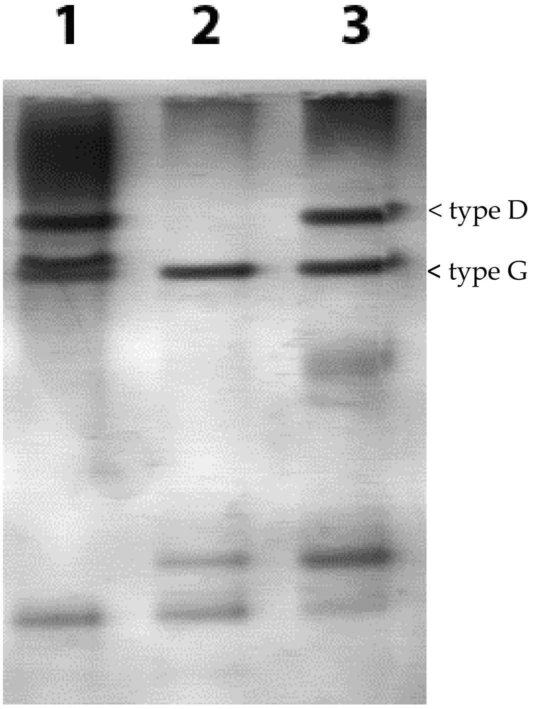

Figure S1. Gelelectrophoretic separation of serum samples from carp belonging to the present study (1), Polish carp showing the type G transferrins (2), and Polish carp containing the allele for transferrin type G and allele D above (3) according to Ilgiz Irnazarow, Institute of Ichthyobiology and Aquaculture, Polish Academy of Science, Golysz, Chybie, Poland.
